# Biobanking, consent, and commercialization in international genetics research: the Type 1 Diabetes Genetics Consortium

**DOI:** 10.1177/1740774510373492

**Published:** 2010-08

**Authors:** Mark A Hall, Nancy MP King, Letitia H Perdue, Joan E Hilner, Beena Akolkar, Carla J Greenbaum, Catherine McKeon, the T1DGC

**Affiliations:** ^a^Division of Public Health Sciences, Wake Forest University Health Sciences, Winston-Salem, NC, USA, ^b^Department of Biostatistics, School of Public Health, University of Alabama at Birmingham, Birmingham, AL, USA, ^c^Division of Diabetes, Endocrinology and Metabolic Diseases, National Institute of Diabetes and Digestive and Kidney Diseases, National Institutes of Health, Bethesda, MD, USA, ^d^Benaroya Research Institute, Virginia Mason, Seattle, WA, USA

## Abstract

***Background and Purpose*** This article describes several ethical, legal, and social issues typical of international genetics biobanking, as encountered in the Type 1 Diabetes Genetics Consortium (T1DGC).

***Methods*** By studying the examples set and lessons learned from other international biobanking studies and by devoting considerable time and resources to identifying, addressing, and continually monitoring ethical and regulatory concerns, T1DGC was able to minimize the problems reported by some earlier studies.

***Conclusions*** Several important conclusions can be drawn based on the experience in this study: (1) Basic international standards for research ethics review and informed consent are broadly consistent across developed countries. (2) When consent forms are adapted locally and translated into different languages, discrepancies are inevitable and therefore require prompt central review and resolution before research is initiated. (3) Providing separate ‘check-box’ consent for different elements of a study creates confusion and may not be essential. (4) Creating immortalized cell lines to aid future research is broadly acceptable, both in the US and internationally. (5) Imposing some limits on the use of stored samples aids in obtaining ethics approvals worldwide. (6) Allowing potential commercial uses of donated samples is controversial in some Asian countries. (7) Obtaining government approvals can be labor-intensive and time-consuming, and can require legal and diplomatic skills.

## Introduction

The Type 1 Diabetes Genetics Consortium (T1DGC) is an international collaborative project where 34 countries organized into four networks worked toward the common goal of collecting and characterizing individuals with type 1 diabetes in order to develop resources for the purpose of identifying genes that increase (or decrease) an individual’s risk for type 1 diabetes. The basic mechanisms that trigger type 1 diabetes are poorly understood, and T1DGC has also facilitated the study of autoimmunity as a general phenomenon that may be implicated causally in type 1 diabetes. A complete list of the countries that participated in each of the four T1DGC networks can be found on the study website (www.t1dgc.org).

The T1DGC has fostered international collaborative gene identification in type 1 diabetes by (1) conducting research worldwide to ascertain, study, and establish a renewable source of DNA from thousands of families with at least two type 1 diabetic children and two parents (if available); families with one type 1 diabetic child and two parents; and matched pairs of diabetic cases and controls; (2) creating a database for the scientific community with standardized clinical, genetic, and medical history information that would facilitate the search for type 1 diabetes susceptibility genes, and a centralized DNA repository to allow targeted studies of genetic structure and function for type 1 diabetes and other autoimmune diseases; and (3) providing opportunities to extend the results of research to develop methods of risk prediction, prevention, and therapy in the area of type 1 diabetes.

The scale and complexity of this international project, along with its targeted focus on type 1 diabetes and other autoimmune diseases, highlight certain ethical and policy issues that are confronted with increasing frequency both in the field and in scholarship, as large sample repository research becomes more widespread. National biobanks have been established in many countries, and repositories to facilitate research investigating gene–environment interactions, pharmacogenetics, and a wide range of disorders and conditions have been established by the pharmaceutical industry, disease constituency groups, cooperative research groups, the National Institutes of Health (NIH), and even hospital consortia [1]. Numerous attempts to develop consistent and workable policies for biorepositories on a range of important ethical and policy questions, including scope of consent, oversight of future uses, recontact of participants, privacy and confidentiality, and intellectual property considerations are ongoing [2–8]. Our experiences with the Consortium in addressing many of these issues over time and all over the world provide practical examples to help inform biobanking policy and scholarship.

## Methods, results, and discussion

Given the international nature of the Consortium, the T1DGC was constructed around four international networks: Asia-Pacific, Europe, North America, and United Kingdom. At the outset of the study, a 10-person Ethical, Legal, and Social Implications (ELSI) Committee was created with one or two representatives from each network, the Coordinating Center (Wake Forest University Health Sciences), and the two funding agencies (National Institute of Diabetes and Digestive and Kidney Disease (NIDDK) and Juvenile Diabetes Research Foundation). It proved to be essential to have a committee with broad interdisciplinary and international composition. At various times in the study, ELSI issues required not only ethical expertise but also legal and diplomatic skills to resolve. The chair of the ELSI Committee devoted 10% of his time to this function over the course of the study. In part, this intensive engagement was needed to meet in person with researchers in each network to explain the nature and source of U.S.-imposed regulatory requirements dealing with the ethics of research. It was necessary both to reassure researchers in some countries that ethical and privacy safeguards were adequate in the United States and to explain the need for requirements that some international researchers viewed as excessive or arbitrary.

## Basic informed consent requirements

The ELSI Committee began by reviewing consent forms used in similar studies, such as the National Human Genome Research Institute’s Haplotype Map (HapMap) project [9] and the National Heart, Lung, and Blood Institute’s Hemochromatosis and Iron Overload Screening (HEIRS) study [10]. In addition, network representatives surveyed local investigators in various countries to determine if our basic planned approach would be satisfactory or if there were issues that the committee had not considered. A draft consent form was circulated and revised several times, including testing for readability, before final approval by the Steering Committee. The final form (Appendix 1) served as a template that individual researchers could follow in seeking Ethics Committee (EC) or Institutional Review Board (IRB) approval. Additional model forms were developed, based on the initial model, for participants in affected sibling pair and trio families, including parents of minor study participants and assent forms for minor participants in different age groups; later, model forms were developed for case and control participants.

We recognized that consent practices differ around the world, that various ECs and institutions use different formats for consent forms, and that different committees may require that additional elements be added or that certain elements be worded differently. An explanatory document was therefore created to help guide investigators and oversight bodies in revising and reviewing site-specific consent forms (Appendix 2). Certain elements of the template were viewed as ethically essential to research with humans [11–13], to genetic and biobanking research [14–17], and to this study, or were required to be standardized for biobanking purposes.

The template was largely successful, as indicated by its widespread adoption in different countries. One aspect, however, proved somewhat problematic. Following what were considered to be best practices at the time, the template adopted a structured consent format that, at the end, restated each of the four core elements of the study (basic participation, central storage of DNA for future research, creation of cell lines, and recontact for participation in future studies) and that required the participant to signify agreement to each one separately. The alternative to this ‘check-box’ approach would have been simply to signify consent to the whole study as described in the entire consent form. The difficulty presented by the check-box approach was the tendency to look to the recapitulations that accompanied the check boxes, rather than to the fuller statement earlier in the form, for the operative permissions language. Because the check-box language necessarily was a summation rather than a complete restatement, it emphasized the genetic purposes of the study. As a consequence, the check-box did not explicitly restate that ordinary serum samples would be stored for nongenetic studies related to diabetes. The more focused check-box language, and inevitable variations in the translated versions, created some issues for the central repository that had to be resolved with the NIDDK Project Office.

Each researcher and country was allowed to change the model form to meet their special needs and concerns. Indeed, they could have entirely rewritten the consent form if they chose. To avoid the appearance of ethical imperialism and allow the flexibility to accommodate widely varying practices, understandings, and social conditions around the world, the ELSI Committee articulated a set of basic ethical standards that would satisfy the study’s core research ethics requirements, regardless of how they might be expressed in a particular consent form. These were as follows:

1. Each participant must be covered by a written consent voluntarily signed by a person with authority.
2. The consent form must explain the basic nature and purpose of the study in language that participants or their authorized representatives can understand.
3. The form must give specific permission to send blood samples to a regional network repository and a central repository located in the United States for storage and future research related to diabetes or autoimmune disorders. (We felt it important to mention the United States as the final destination due to widespread anti-American sentiment at the time.)
4. The consent form must explain DNA or genetic research in some way. Also, in order for cell lines to be created, the consent form must explain their nature and purpose in some way.

These core requirements presented no substantive problems in any country. Indeed, despite this flexibility to rewrite or write consent forms from scratch, all participating institutions started with the full template and most made only minor adjustments. The only major change was that some institutions declined to use the template’s structure of granting consent separately and specifically, using check-boxes, to each of four elements of the study (described above).

To ensure that basic requirements were met, we asked that revised consent forms be translated back into English; these back-translated consent forms served as the permanent record of their content. The consent forms were then reviewed by NIDDK. This review revealed a number of discrepancies in the critical permission language that arose during the translation and back-translation process – discrepancies that initially escaped the attention of the project’s staff. Consistency in this key language is essential to central storage for future research. For instance, some translated consents gave permission to store and use only DNA, and not plasma and serum, and some failed to mention the use of samples in studying the complications of diabetes or other autoimmune diseases. Some discrepancies introduced conditions or qualifications that were simply unacceptable to the central repository; for instance, a requirement that a local institution approve all future uses.

Several steps were taken to straighten out these problems. Some minor discrepancies were resolved simply by doing a more accurate back-translation or by asking the approving EC for a letter giving a clarifying interpretation that was consistent with the model template. Other discrepancies required the country to revise its approved consent form. Because this review by the central repository was not done at the earliest possible stage, it was necessary in one country to re-consent some participants who had been enrolled prior to correction of the consent forms. A few samples were ultimately destroyed when participants could not be re-consented. Biosample collectors are well advised to anticipate the likelihood of discrepancies of language and meaning, and to ensure that consent form changes and back-translations are reviewed as early as possible by the eventual repository, certainly prior to initiating sample collections.

Although considerable effort was required to adapt and translate consent forms for use in multiple study sites in many countries, in many respects, these challenges were quite similar to those faced in any large multicenter study [18]. The IRBs at many sites request changes, and a coordinated review of these changes is required to ensure that local preferences are balanced appropriately with the requirements of the study. Although this labor-intensive process is criticized with some justification in single-country studies, it is essential in multi-country studies. Fortunately, our experience suggests that centralized review of local changes to consent forms is workable and can indeed achieve an effective balance.

## Creation of cell lines

Collected samples were used to create immortalized cell lines. Investigators were required to tell participants that a cell line would be created unless they objected, because it was felt that some people might oppose having an ongoing means to produce more of their DNA indefinitely, rather than only the quantity of DNA that is extracted initially. The following template language was used by most sites: ‘To allow more researchers to work with your blood sample, we are requesting permission to produce and store a living cell line, which means we will keep some of your white blood cells alive for future research. If you agree, this will give researchers a large supply of DNA without needing to draw additional blood samples.’

Nevertheless, people were given the option of participating even if they objected to creating a cell line. The other option would have been simply to exclude objectors from the study, but there was concern originally that this would make recruitment more difficult. However, less than 1% of participants in the Asia-Pacific, European, and United Kingdom Networks exercised the option to object to cell lines, but over 3% did so in the North American Network. This suggests that concerns about immortalized cell lines are low and are much lower elsewhere in the world than they are in the United States.

## Uses of samples and information

The purpose of this study and future studies was stated somewhat broadly to include the ‘complications’ of diabetes and ‘other autoimmune diseases.’ It would not be useful to store samples in a central repository unless permission for a reasonable range of related studies is given, because it is not feasible to re-contact participants in more than 34 countries or research groups each time a particular study is proposed in the future.

The optimal scope of participants’ consent to uses of biospecimens in future studies is one of the liveliest controversies in genetic and biobanking research [1–8,14–17]. Although many investigators, scholars, and policy makers regard blanket consent as the best means of promoting research progress, others maintain that a narrower scope of research makes participants’ consent more meaningful and helps to guide oversight bodies’ review of future proposed research.

To protect the rights of participants and the Consortium’s purposes, other researchers are given access only if they are qualified and they propose to do relevant studies. In general, these requirements mean that all investigators seeking to use T1DGC samples and data must have their studies reviewed and approved by an EC or IRB; limit their studies to the purposes of the Consortium; provide information about the investigators and their affiliations, their funding sources, and the potential medical, scientific, and commercial applications of the research; ensure security of samples; agree not to share or distribute samples; and agree to destroy samples when work is done.

Contributing investigators, in order to pursue their own research, received DNA and a cell line from each participant's sample they contributed to the study. The Consortium therefore considered how investigators may use these retained samples collected from their own participants. In addition to restricting use to the study of type 1 diabetes, its complications, and other autoimmune diseases, the following policies were adopted:

1. If participants request to withdraw from the T1DGC study, contributors must be able to destroy their samples and information, including any samples or information that the investigator might give to their collaborators.
2. Contributors may not (unless permitted by an IRB or EC) attempt to identify their samples or link them to information that could identify the participants they came from, because they were collected under the understanding that they would be stored and studied anonymously.
3. Contributors who wished to pursue other lines of research, or make other uses of samples beyond these restrictions, could do so only by collecting additional samples outside of this study, using a separate and different informed consent.

## Commercial uses

Intellectual property considerations are of increasing importance in all areas of biotechnology research, including genetic research [19–22]. The debate over whether scientific progress is best fostered by awarding patent rights or preserving open access is ongoing. Information-sharing is mandated by many research funders, prompting the need to address the possibility of ‘downstream’ commercial uses of study data.

The philosophy of this study was to make available as much information as possible, to as many qualified researchers as possible, in order to improve the health of people affected by type 1 diabetes. Therefore, this study placed its samples and data in the public domain for the benefit of science and medicine and did not claim any intellectual property rights (other than rights to control use and access to the database and biobank).

However, it is possible that other researchers or companies who have access to these public domain materials may be able to use them to develop something with commercial value or to claim intellectual property in some product of their research using these materials. The only way to prevent such commercial uses and claims would be to permit research access to only governments or nonprofit organizations, but that would weaken the purposes of the Consortium.

These policies allowing possible downstream commercialization were acceptable in Europe, North America, United Kingdom, and much of the Asia-Pacific region, but several Asian countries were concerned about commercial uses of their samples. These countries were permitted, if they wished, to flag their samples in the database as not available to profit-making companies or for researchers who intend to pursue commercial development of their discoveries. Only India ended up insisting on flagging their samples this way. For Thailand, it was necessary to state that the samples remained the property of the collecting institution, even when they were sent to the United States for central storage. This stipulation was deemed acceptable because it did not interfere with any of the Consortium’s or central repository’s policies about storage, use, and disposal.

## Reporting results

Reporting the results of genetic research has become another topic of considerable scholarly and policy interest [14,23,24], in part because of high expectations that the discovery of genetic associations may lead rapidly to the development of effective treatments, and in part because of the difficulty in reconciling the limited value of complex genetic information in clinical applications with its inherent meaning for individuals. Thus, whether or not to report the results of studies like T1DGC is important both to determine and to explain to all involved.

The Consortium did not report any results directly to participants; it did only to investigators. Each investigator was allowed to decide whether to report any results to participants. Some investigators decided to report human leukocyte antigen (HLA) genotyping or autoantibody test results in the event that this information might be helpful for refining clinical diagnoses or treatment plans. Also, investigators always had the option of recontacting participants to notify them of the option for additional testing (without study funding) in the event that subsequent research reveals genetic information that has clinical importance. Updates on the progress of the Consortium, its findings, and its publications are reported on the T1DGC website, which participants can access if interested.

## Government approvals

Any institution that receives federal funding for human subjects research must have a ‘Federal Wide Assurance’ (FWA) issued by the Department of Health and Human Services' (DHHS) Office of Human Research Protections (OHRP). This FWA requires a functioning EC that resembles the US’s IRB and assurance of compliance with various basic ethics standards. It was correctly anticipated that it would be a burden to require investigators in far-flung parts of the world to satisfy these requirements on their own. Initially, it was hoped that this requirement could be avoided by paying local investigators only through funds that the four multi-country networks administered, out of institutions with FWAs in Melbourne, Australia; Copenhagen, Denmark; Seattle, WA, USA; and Cambridge, United Kingdom, rather than enabling investigators to receive funds directly from NIH. However, the DHHS ruled that each investigator must affiliate with a local institution that has an FWA. Therefore, staff in the four Network Centers were trained in assisting investigators with using the OHRP’s website to meet this requirement. Also, to ease this burden, especially for investigators at institutions without functioning ECs, investigators were allowed to affiliate with institutions in their country that had existing FWAs as long as that institution’s EC agreed to assume oversight responsibility.

Another regulatory issue that had to be negotiated was the need in the United States to satisfy the requirements of the Health Insurance Portability and Accountability Act (HIPAA) privacy rule [25], in order to send data to Consortium participants. Similarly, the European Commission’s directive on personal data privacy sets forth protections that institutions in other countries must show before they can receive personal medical data from European countries [26]. To meet the European requirements, the Coordinating Center had to obtain certification for safe harbor status (similar to the US’s FWA). To meet HIPAA requirements, the Coordinating Center’s legal office initially expected that we use its standard ‘data use agreement’, but project leaders were concerned that it would alienate contributing investigators if the United States imposed yet another set of demanding regulatory requirements, especially ones containing threatening legalese.

The solution devised was to redraft the data use agreement to consist of a set of reciprocal assurances between the Consortium and its members, in a fashion that would satisfy both HIPAA’s requirements and those of the European Privacy Directive, and therefore presumably also the laws of other countries. In other words, rather than creating one set of agreements under European law that covered the sending of information to the Coordinating Center, and a second set of agreements under US law that covered the return of information to local investigators, a single agreement was written that served both purposes (Appendix 3). The reciprocity created by putting these mutual assurances in the same agreement helped to ease possible objections to US legal and ethical imperialism. Also, it was determined that this agreement needed to be signed only by the Coordinating Center and the four Network Centers, and not by individual contributing investigators in each network. These strategies worked well.

India and Thailand required approval by a central government agency before local investigators could participate, and before blood and DNA samples could be sent abroad. Their concern is that national resources will be exploited or expropriated for financial gain elsewhere, without commensurate benefit for their citizens. Sometimes (as in Thailand), government approval simply required reviewing and revising the terms of a ‘material transfer agreement’. Other times, this hurdle proved to be considerable. In India, the process of preparing the extensive proposal forms and shepherding them through the governmental review process consumed over 3 years – threatening the feasibility of that country’s full participation.

## Conclusions and recommendations

As a long-term, large-scale international study, T1DGC has dealt with most of the major ethical and policy issues associated with biobanking, and indeed has seen several ‘best practices’ change over time. The Consortium faced fewer problems than those reported by some other, similar studies [27,28]. The Consortium’s success in identifying and addressing ethical concerns did not come easily but stems from several key strategies: (1) dedicating sufficient time, personnel, and resources to ELSI issues; (2) devoting hands-on attention to good communication, in order to ensure that concerns are understood and solutions are responsive and implementable; (3) being able to persist, redo, revise, and revisit in order to ensure that solutions are implemented; and (4) following up continually, and expecting new issues to arise over time.

Based on the experience in this study, several important conclusions can be drawn as follows:

1. Basic international standards for research ethics review and informed consent are broadly consistent across developed countries.
2. When consent forms are adapted locally and translated into different languages, discrepancies are inevitable and therefore require prompt central review and resolution before research is initiated.
3. Providing separate ‘check-box’ consent for different elements of a study creates confusion and may not be essential.
4. Creating immortalized cell lines to aid future research is broadly acceptable, both in the United States and internationally.
5. Imposing some limits on the use of stored samples aids in obtaining ethics approvals worldwide.
6. Allowing potential commercial uses of donated samples is controversial in some Asian countries.
7. Obtaining government approvals can be labor intensive and time consuming, and can require legal and diplomatic skills.

Although national, cultural, and language differences gave rise to many of the ethical issues encountered by T1DGC, many issues commonly arise in similar multicenter studies conducted entirely within the United States or Europe. Thus, T1DGC has been something of a bellwether for ethical issues in biobanking and genetic research. Because the best ethics are preventive, we hope that what we have learned can help others anticipate and preemptively address these and similar issues in their own research.

## Appendix 1

### Model Informed Consent Form

*[To be modified to meet requirements of Networks and Ethics Committees. Words in [brackets] are optional and sentences in italics are instructions. These should be deleted in the final version of this form. It is permissible to change the order of sentences or paragraphs. Other changes also may be permissible, but they should be discussed with the Network first. The final version of this form must be translated back into English and sent to the Regional Network Center.]*

Type 1 Diabetes Genetics Consortium

Sponsor: United States National Institutes of Health (NIH), National Institute of Diabetes & Digestive & Kidney Diseases (NIDDK)

[Name of Local Institution]: ——————————

You are invited to participate in the Type 1 Diabetes Genetics Consortium. This is an international effort to identify genes that affect the risk of type 1 (or juvenile) diabetes. The researchers listed below are in charge of this study at this institution. Other staff will help or act for them, and they are working with other researchers around the country and the world who are also part of this study.

Before you can decide whether or not you should agree to join this study, you should learn about its risks and benefits. This is called informed consent. If you decide to join the study, you will sign this Informed Consent Form and you will have a copy to keep for your records.

### Purpose of the genetic study

Type 1 (or juvenile) diabetes is an important health problem that affects many people. Doctors know that genes (DNA) play a major role in type 1 diabetes. This means that the risk for developing type 1 diabetes can run in a family and be passed from parents to children. Recent advances in science allow doctors to study the genes in families of people with a disease to learn more about which genes affect the risk of the disease. This can help to develop better treatments or prevent health problems, which is why we are conducting this study. However, to do this, lots of different families are needed to provide samples of their blood to analyze their genes (DNA). More detailed information on this study is available on the web site: www.t1dgc.org.

### Description of the study

If you agree to be part of this study, we will take 3 to 5 tubes of blood (about 3 soup spoons [or 39 mL]) from your arm and process this sample so that DNA and other parts of your blood can be taken out, stored, and used for research. We will also ask you some questions about your health and your family. This is being done for our research purposes only. You will not learn anything through this research about yourself or your family members. *[Or: describe policy for reporting results.]*

With your permission, your blood samples and information will be stored by —————*[specify local clinic]* and also sent to storage locations in —————— *[specify regional network storage sites]* and a central repository in the United States so they can be shared with other qualified researchers and companies worldwide to study the genetics of type 1 diabetes, its complications, and other autoimmune diseases. These researchers may or may not be part of this study. Because of the sensitive nature of genetic materials, guidelines described below have been developed to protect your privacy.

*[Delete this sentence if there is a policy to report some results:]* You will not receive any information from us after we take your blood sample*.* However, we might ask for your help in contacting your family members to participate in this study, and we might contact you again for permission to collect additional samples or information.

### Consent to produce a cell line

To allow more researchers to work with your blood sample, we are requesting permission to produce and store a living cell line, which means we will keep some of your white blood cells alive for future research. If you agree, this will give researchers a large supply of DNA without needing to draw additional blood samples. We will allow other researchers to use your stored sample only to study type 1 diabetes, its complications, and other autoimmune diseases. [We will not do or allow any human cloning.]

### Ownership and right to have genetic material destroyed

When you give your blood sample, you will no longer own it or the genetic materials we obtain from it. However, you have the right to request at any time that your blood sample and genetic materials be destroyed. Your request will be honored and we will tell you when your samples have been destroyed.

### Risks and discomforts

There are no major risks associated with drawing blood. Having your blood drawn can be uncomfortable, occasionally causes bruising, and, in rare cases, causes fainting. Only trained people will draw your blood. There is also a very small risk that some breach of confidentiality may occur. The specific protections put in place to prevent this are discussed in the confidentiality section of this form.

### Benefits

There are no direct benefits to you from this study. By participating in this study, you may or may not help doctors develop better treatment or prevention for type 1 diabetes, its complications, and other autoimmune diseases. Throughout the study, research findings will be posted on the web site: www.t1dgc.org.

### Alternatives and right to withdraw from the study

Participating in this study is entirely up to you. You may refuse to participate or withdraw from the study at any time, and this will not affect your current or future health care or other benefits at *[Name of Institution]*.

### Confidentiality

We will keep all of your medical and genetic information confidential to the extent the law allows. However, we cannot guarantee absolute confidentiality. Information about you will not be given to insurance companies, your employers, or be used for any purposes other than those described in this agreement. The information from this research will be made widely available to researchers, doctors, scientists and other people, but your identity will not be released.

Your blood and DNA samples and the information you give us will be stored in different places under a code number, without your name or other identifying information. However, we will still be able to identify you if we need to for research reasons.

Because other family members are participating in this study, it is possible that we may find out personal information that you or your relatives do not know or do not want others to know [(for example, that someone’s biological father is not who they thought it was)]. If we discover this, we will not tell you or anyone in your family under any circumstances.

In some cases, people from the government agency that is paying for this research, the U.S. National Institute of Diabetes and Digestive and Kidney Diseases (NIDDK), may need to see your records to verify study information, but they will not be told who you are. Also, the ————— *[name of institution's Ethics Committee/IRB*] may have access to your records to ensure that your rights are being properly protected.

### Costs, compensation, and treatment

There is no charge to you for being in this study. You will also not receive any payment for being in this study. [Or: We will pay you $—— to cover your travel or other expenses for being in this study.] Your blood, DNA, and information will not be sold. However, it is possible that information or materials from this study might be used to develop products that have commercial value. If this happens, you will not receive any share of the profits. We will not give you any treatment as part of this study.

### Questions

If you have any questions about your rights as a research participant, please phone *[include name of Ethics Committee/IRB representative if appropriate]* of the Ethics Committee [Institutional Review Board], at *[insert phone number]*. Moreover, if you have any questions or concerns regarding this study, or if problems arise, you may call the Principal Investigator, *[insert name],* at *[insert phone number]*.

### Participant’s statement

I understand that, by participating in this study, I agree:

A. To give 3 to 5 tubes of blood for storage, processing, and research on the genetics of type 1 diabetes, its complications, and other autoimmune diseases; to have blood tests done relating to diabetes if needed; and to answer questions about my health and my family, understanding that this information will be kept confidential at all times and that I can ask to have my samples destroyed at any time.

□ Yes

B. And, to allow my information and the DNA extracted from my blood samples to be sent to the United States and given to other qualified scientists worldwide, even after this study ends, to be analyzed for genetic information relating to type 1 diabetes, its complications, and other autoimmune diseases.

□ Yes

I also agree:

C. To allow my genetic material to be made into a living cell line that will create an unlimited supply of DNA that can be used to study type 1 diabetes, its complications, and other autoimmune diseases.

□ Yes** **□ No

D. To be contacted in the future about possibly participating in additional studies related to diabetes, its complications, and other autoimmune diseases.

□ Yes** **□ No

### Signatures

Your signature below shows that you have read this consent form and agree to join this study.

_______________Date:_______________

Signature

_______________Date:_______________

Signature of Witness

Type 1 Diabetes Genetics Consortium Study Investigators:

*[include names, addresses, phone numbers]*

## Appendix 2

### Explanation of Informed Consent Form

#### General comments

Because of the sensitivity of genetic information and the complexity of a world-wide study, the consent form used for T1DGC may be longer than those used in clinical studies. Consent must be signed and in writing. Each participant should either consent or have someone with authority, such as a parent or guardian, consent for them. Parents may sign a consent form that covers more than one child. If children are able to understand the nature and purpose of the study, they can also be given a consent form, or a simple explanation can be read to them. This is called ‘assent’ rather than ‘consent.’ For this purpose, there are two versions of the assent form, one for small children and the other for older children.

Each researcher and country may change the model form to meet their special needs and concerns. However, changes to the substance of the form must be approved by the Network. Consent forms must be translated into a participant’s native language. Translated versions of the forms in other languages must be back-translated into English to have a permanent record of the content of the consent form.

#### Amount of blood drawn

Only three tubes will be drawn on children less than 16 years old (22 mL) and four tubes on individuals who are 16 years or older (32 mL). Five tubes (an additional 5 or 7 mL) will be drawn only occasionally, for a quality assurance check, and only from participants who are 16 years or older. In the case of very small infants, the blood draw may need to be less if local guidelines do not permit three tubes to be drawn (based on the child’s weight or total blood volume).

#### Reporting results

The Consortium will not report any results to participants, only to investigators. Each investigator or ethics committee may decide whether to report any results to participants. Most investigators will not report results because it is not expected that testing will produce any clinically important information. Antibody testing is being done only on people already diagnosed with diabetes, not on any unaffected family members. In its genetic analysis, the Consortium expects only to identify regions that require more detailed study.

Some investigators or ethics committees may decide that they want to report HLA genotyping or antibody test results. If so, they need to decide whether these results will be reported to all participants, or only to those where the results show elevated risk or something the participant or their physician is not already aware of. Investigators should consider the burden (on themselves and on participants) of explaining test results where clinical importance is not clear or where participant anxiety may occur but there is no clear course of action or treatment. If an investigator decides to report some or all results, this policy needs to be described accurately in the consent form.

It is possible that, in the future, particular researchers could use Consortium resources to identify a specific gene that identifies susceptibility to diabetes, but that possibility is too uncertain to offer as a potential benefit of the study. If this were to happen, investigators always have the option of recontacting participants to notify them of the possibility for additional testing, but this will need to be paid for from sources outside of this study.

#### False paternity

The model form suggests telling participants that they will not be notified if some indication is found ‘that someone’s biological father is not who they thought it was.’ This is a study policy that cannot be changed, in part because indications of possible false paternity will not be conclusively confirmed. However, it is optional whether or not to notify participants specifically about this policy. Some institutions may prefer not to raise this issue.

#### DNA and cell lines

It is necessary to tell participants that a cell line will be created, because some people may object to this. However, people can still participate in the study if they refuse permission for a cell line, but then researchers will be limited to working with only the quantity of DNA that is extracted initially. Creating a cell line promotes the interests of research participants because it reduces the amount of blood that it is necessary to remove. This is particularly important for studies like this that involve small children or infants. It has sometimes happened in the past that genetic studies have exhausted the stored DNA samples before the study is successfully completed, but when success is still possible. This defeats the purpose of asking people to participate and to donate their samples.

The model form explains that a cell line is not the same as human cloning and that no human cloning will be done or will be allowed by this study. This policy will not change. However, it is optional whether to include this language that specifically raises this issue, or instead to only explain this if a person asks about the issue of cloning.

#### Future studies

DNA and information will be stored and released to other researchers only in a strictly anonymous form. Also, this will be done only for diabetes-related studies by qualified researchers that are approved by ethics committees (IRBs) and that meet the requirements established by the Consortium; these are described further below.

The purpose of this study and future studies is stated somewhat broadly to include “other autoimmune diseases” because the basic mechanisms that trigger type 1 diabetes are poorly understood, and it may be important to study autoimmunity as a general phenomenon in order to understand what causes type 1 diabetes. Also, the consent form allows future studies of the ‘complications’ of diabetes. However, the actual range of complications or autoimmune diseases that is feasible to study is limited to the particular conditions that participants are asked about in the health questionnaires. These include multiple sclerosis, celiac disease, thyroid disease, myasthenia gravis, pernicious anemia, lupus, rheumatoid arthritis, inflammatory bowel disease, vitiligo, Addisons disease, and psoriasis. The DNA samples are not useful for a central repository if this permission is not given, because it is not feasible to re-contact participants in 34 countries or research groups each time a particular study is proposed in the future.

#### Commercial uses

The philosophy of this study is to make available as much information as possible, to as many qualified people as possible, in order to improve the health of people affected by this devastating disease. The Consortium was established because leading researchers believed that existing collections of DNA samples were too small and kept by separate researchers. Therefore, this study intends to pool all available DNA and place it in the public domain for the benefit of science and medicine. The Consortium will not claim any intellectual property rights in this DNA, it will not sell the DNA, and it will not, itself, develop any commercial products from this DNA or the related information.

However, it is possible that other researchers or companies who have access to these public domain materials may be able to use them to develop something with commercial value. The DNA itself will not be commercialized; it is only possible that the DNA might be raw material that is used to develop a commercial application. The only way to prevent this is to deny access to the materials, both to scientists and to companies, but that would defeat the purposes of the Consortium.

To protect the rights of participants and the Consortium’s purposes, other researchers will be given access only if they are qualified and they propose to do relevant studies. Exact requirements for qualification are still being developed by the Consortium’s Access Committee. In general, the Consortium will require that other researchers and companies:

- Honor the Consortiums’ rules about ownership and intellectual property
- Have their studies approved by Ethics Committees (IRBs)
- Limit their studies to the purposes of the Consortium
- Provide detailed information about the purpose and nature of the study, the funding sources, the investigators and their affiliations, the potential medical, scientific, and commercial applications of the research, and other relevant considerations
- Ensure security of samples, do not share or distribute samples, and destroy samples when work is done
- Sign a pledge form to abide by T1DGC publications policies, the consent guidelines conferred by study participants, and T1DGC policies related to ownership of specimens and patent rights

#### Compensation

It is optional whether or not to compensate participants, but if there is any compensation, it should only be for expenses, not as payment to ‘purchase’ participation. Institutions are free to modify the suggested language to fit the circumstances of their particular compensation policies.

[North American Version Only:]

#### Confidentiality

The section of confidentiality, and language elsewhere in the form, is intended to comply with the new U.S. privacy law known as HIPAA, which requires a detailed explanation of where data is kept and who has access to it. Also, HIPAA requires that certain other statements be included that might appear unusual or unnecessary. With these provisions in the consent form, it is not necessary to have a separate HIPAA ‘authorization form’ signed, which would repeat what is already in the consent form.

## Appendix 3

### Data Use Agreement

This **DATA USE AGREEMENT (‘the Agreement’)** is effective the ——day of —————, 2006, by and between **Wake Forest University Health Sciences (‘Wake Forest’),** and —————— (‘**Participant’**).

### RECITALS:

**WHEREAS,** Wake Forest and Participant are part of the Type 1 Diabetes Genetics Consortium (the ‘**Consortium’**), whose general purpose is to identify genes that determine an individual’s risk of type 1 diabetes;

**WHEREAS,** the Consortium requires the transfer, sharing, analysis, and other uses of various types of medical data among the many institutions that are participating internationally;

**WHEREAS,** medical data is regulated by various laws, regulations, protocols, and guidelines in both the United States and in other countries that are part of the Consortium, which the parties to this agreement wish to comply with;

**NOW, THEREFORE,** in consideration of the mutual agreements, covenants, terms and conditions herein contained, Wake Forest and Participant agree as follows:

### I. Transfer and use of limited data

Section 1.1 **Activities.** Wake Forest and Participant will use and transfer data under this agreement only for the research purposes of the Consortium, specified in its Consortium Agreement, its Statement of Purposes, and its Specific Aims, as they may be modified from time to time.

Section 1.2 **Limited Data.** Wake Forest and Participant will transfer to each other data that is collected through the forms and protocols established by the Consortium, as they may be amended from time to time. Wake Forest and Participant acknowledge that these data elements are the minimum necessary for accomplishing the research purposes of the Consortium. Data that are transferred between Wake Forest and Participant will not contain any of the following information that can be used to identify the research participant or their relatives, household members or employers: names, telephone numbers, fax numbers, street addresses, electronic mail addresses, social security numbers, medical record numbers, insurance identification numbers, account numbers, certificate/license numbers, vehicle identifiers and serial numbers (including license plate numbers) device identifiers and serial numbers, Web Universal Resource Locators (URLs); Internet Protocol (IP) address numbers, biometric identifiers, full face photographic images and comparable images. Data without these personal identifiers shall be known as ‘**Limited Data**.’

Section 1.3. **Use of Limited Data.** Wake Forest and Participant may use and disclose the Limited Data only as permitted under the terms of this Agreement or required by law, but shall not otherwise use or disclose the Limited Data and shall ensure that its directors, officers, employees, contractors and agents do not use or disclose the Limited Data in any manner that would constitute a violation of this Agreement or applicable law. Wake Forest and Participant agree not to use the Limited Data in such a way as to identify any individual and further agree not to contact any individual who might be identified using this data. Data User shall limit the use or receipt of the Limited Data to the individuals who reasonably need the Limited Data for the performance of the Consortium’s Activities*.* Wake Forest and Participant shall use appropriate safeguards to prevent use or disclosure of the Limited Data other than as permitted under this Agreement.

Section 1.4. **Reporting of Disclosures of Protected Health Information.** Wake Forest or Participant shall, within thirty (30) days of becoming aware of any use or disclosure of the Limited Data in violation of this Agreement, report any such use or disclosure to the other party to this Agreement.

Section 1.5. **Notice of Request for Data**. Wake Forest and Participant agree to notify the other party within (7) business days if it receives an official request or legal subpoena for any Limited Data. To the extent that one party decides to challenge the validity of such request, the other party shall cooperate fully in such challenge.

Section 1.6. **Agreements by Third Parties.** Wake Forest and Participant shall obtain and maintain an agreement with each agent or subcontractor that has or will have access to the Limited Data, which requires such agent or subcontractor to be bound by the same restrictions, terms and conditions that apply to the Limited Data under this Agreement.

### II. General provisions

Section 2.1. **Termination Upon Breach.** Any other provision of this Agreement notwithstanding, this Agreement may be terminated by either party upon fifteen (15) days written notice (including e-mail) to the other party in the event that the second party breaches any provision contained in this Agreement and such breach is not cured within such fifteen (15) day period. Wake Forest and Participant acknowledge and agree that in the event the other’s efforts to cure any breach are unsuccessful, the first party has a duty to discontinue use of the Limited Data, notwithstanding any other provision of this Agreement to the contrary.

Section 2.2. **Return or Destruction of Data upon Termination.** If this Agreement is terminated due to breach of the Agreement, the breaching party shall either return or destroy all data received from the other party that the breaching party maintains in any form. The breaching party shall not retain any copies of such data. Notwithstanding the foregoing, to the extent that the non-breaching party agrees that it is not feasible to return or destroy such data, the terms and provisions of this Agreement shall survive termination of the Agreement and such data shall be used or disclosed solely as required by the reasons that prevented their return or destruction.

Section 2.3. **Injunction.** Wake Forest and Participant each acknowledge and agree that the other party will suffer irreparable damage if it breaches this Agreement and that damages from such breach shall be difficult to quantify. Therefore, Wake Forest and Participant acknowledge and agree that an action for an injunction may be filed to enforce the terms of this Agreement, in addition to any other remedy the law may provide.

Section 2.4. **Effect.** The terms and provisions of this Agreement shall supercede any other conflicting or inconsistent agreements between Wake Forest and Participant, including all exhibits or other attachments thereto and all documents incorporated therein by reference.

**IN WITNESS WHEREOF,** the parties have caused this Agreement to be executed as of the day and year first written above.

**Wake Forest:      Participant:**

By:________________ By:_______________

Title:_______________ Title:_______________

Date:_______________ Date:_______________
